# Mid-Embryo Patterning and Precision in *Drosophila* Segmentation: Krüppel Dual Regulation of *hunchback*

**DOI:** 10.1371/journal.pone.0118450

**Published:** 2015-03-20

**Authors:** David M. Holloway, Alexander V. Spirov

**Affiliations:** 1 Mathematics Department, British Columbia Institute of Technology, Burnaby, B.C., V5G 3H2, Canada; 2 Computer Science, and Center of Excellence in Wireless and Information Technology, State University of New York, Stony Brook, Stony Brook, New York, United States of America; 3 The Sechenov Institute of Evolutionary Physiology and Biochemistry, St. Petersburg, Russia; Imperial College London, UNITED KINGDOM

## Abstract

In early development, genes are expressed in spatial patterns which later define cellular identities and tissue locations. The mechanisms of such pattern formation have been studied extensively in early *Drosophila* (fruit fly) embryos. The gap gene *hunchback* (*hb*) is one of the earliest genes to be expressed in anterior-posterior (AP) body segmentation. As a transcriptional regulator for a number of downstream genes, the spatial precision of *hb* expression can have significant effects in the development of the body plan. To investigate the factors contributing to *hb* precision, we used fine spatial and temporal resolution data to develop a quantitative model for the regulation of *hb* expression in the mid-embryo. In particular, modelling *hb* pattern refinement in mid nuclear cleavage cycle 14 (NC14) reveals some of the regulatory contributions of simultaneously-expressed gap genes. Matching the model to recent data from wild-type (WT) embryos and mutants of the gap gene *Krüppel* (*Kr*) indicates that a mid-embryo Hb concentration peak important in thoracic development (at parasegment 4, PS4) is regulated in a dual manner by Kr, with low Kr concentration activating *hb* and high Kr concentration repressing *hb*. The processes of gene expression (transcription, translation, transport) are intrinsically random. We used stochastic simulations to characterize the noise generated in *hb* expression. We find that Kr regulation can limit the positional variability of the Hb mid-embryo border. This has been recently corroborated in experimental comparisons of WT and *Kr-* mutant embryos. Further, Kr regulation can decrease uncertainty in mid-embryo *hb* expression (i.e. contribute to a smooth Hb boundary) and decrease between-copy transcriptional variability within nuclei. Since many tissue boundaries are first established by interactions between neighbouring gene expression domains, these properties of Hb-Kr dynamics to diminish the effects of intrinsic expression noise may represent a general mechanism contributing to robustness in early development.

## Introduction

In the early stages of *Drosophila melanogaster* segmentation, *hunchback* (*hb*) and other gap genes form broad expression domains along the anterior-posterior (AP) axis of the embryo. Their transcription is regulated by maternally-derived proteins and by cross- and self-effects of the gap proteins themselves. Maternal and gap factors have been the focus of many quantitative studies on how spatial gene expression patterns form. *hb* activation by the anterior-high maternal protein Bicoid (Bcd), in particular, has served as a model system for studying positional specification by the concentration of a spatially-graded factor [1, 2]. The degree to which *hb* is characterized, from sequence-level information of its cis-regulatory regions to the temporal and spatial dynamics of its expression (and of its transcriptional regulators), allows for the development and testing of detailed quantitative (mathematical) models of the different factors (e.g. maternal, self-, and cross-regulatory) affecting developmental patterning. Since the Hb protein itself forms a spatial gradient controlling several expression boundaries of downstream segmentation genes [3, 4] in nuclear cleavage cycle 14 (NC14; the syncytial blastoderm stage), precise control of its expression is an early component in the robust development of fruit flies, as well as other insects [5–7].

One of the key developmental questions studied with *hb* is how gene expression patterns maintain reliability despite extrinsic sources of variability (in global factors such as embryo size and regulatory factor concentrations) and intrinsic noise (due to the inherently random nature of gene expression, seen, for example, in transcriptional bursting). Extrinsically, comparisons of Hb and Bcd positional variability in the mid-embryo indicate that mature NC14 *hb* pattern is not entirely specified by Bcd concentration [8–10]: rather than a one-to-one correspondence, Hb displays about half the variability of Bcd [11]. This means that the spatial precision that *hb* (and other gap genes) provide to their targets, the pair-rule genes, is about twice the precision supplied to the gaps by the maternal gradients [12]. This increase in precision at the gap level is likely due to a number of factors, including gradient reading and synchronization prior to NC14 [13–15], as well as gap-gap interactions during NC14 (e.g. [16,17]). Intrinsically, advances in imaging have made *hb* a focus for studying gene expression noise within embryos—characterizing the degree to which expression differs between nuclei spatially and temporally, and investigating the sources of this variation [18–21]. This paper focuses on ways in which gap-gap interactions affect the intrinsic noise of *hb* expression.

**Fig 1:**
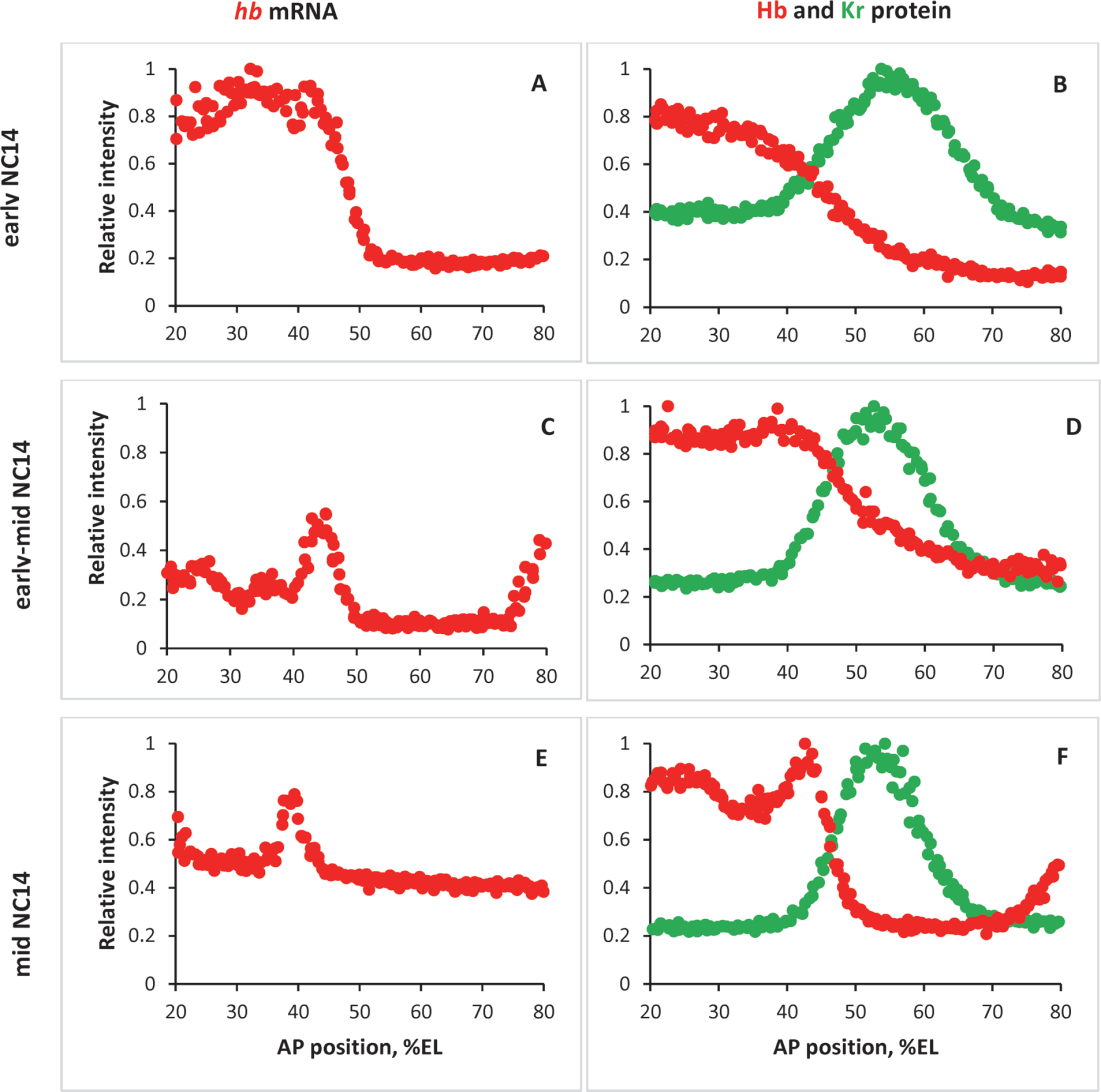
Maturation of *hb* expression patterns in NC14. Data from whole embryos staged, fixed and stained for segmentation gene products, from the BDTNP database (http://bdtnp.lbl.gov/Fly-Net/bioimaging.jsp). Plots show fluorescence intensity (proportional to concentration) on the vertical axis (normalized to maximum intensity for each signal) against the anterior (left)—posterior (right) axis on the horizontal (in relative units of percent egg length, %EL). Nuclear intensities (each dot) are from a 10% dorsoventral (DV) strip along the lateral midline of the embryo. (A) *hunchback* (*hb*) mRNA, NC14 onset (embryo 12781-29fe08-22; 0% membrane invagination). (B) Hb (red) and Krüppel (Kr, green) protein, NC14 onset (12120-17se07-17, 0% invagination). (C) *hb* mRNA, early-mid NC14 (10541-18mr05-32, 20% invagination). (D) Hb (red) and Kr (green) protein, early-mid NC14 (12057-20jn07-12, 20% invagination). (E) *hb* mRNA, mid NC14, in the MBT (10875-27de05-05, 40% invagination). (F) Hb (red) and Kr (green) protein, mid NC14, in the MBT (Hb: 12824-19mr08-23, 42% invagination; Kr: 12057-20jn07-08, 40% invagination). (A, B) show the early, smooth, Bcd-dependent *hb* profiles; (C, D) show the transition to the sharper, peaked expression established by mid NC14 (E, F).

At early NC14, *hb* expression is largely Bcd-dependent, forming a broad anterior domain (Fig. 1AB). Bcd-dependent *hb* transcription shuts off shortly into NC14 [22]. Within about 15 minutes into NC14, *hb* self-activation becomes important for sharpening the Hb mid-embryo boundary [23,24]. By mid-NC14, other gap genes are also being strongly expressed, and gap cross-regulation becomes increasingly important in shaping Hb and the other gap domains e.g. [16,17,25–32].

A distinct feature of mid-NC14 is the refinement of gap patterns into peaks or sub-domains as the embryo approaches the mid-blastula transition (MBT), when zygotic expression becomes dominant. Fig. 1 shows the change from simpler early *hb* pattern (Fig. 1AB) to later pattern with distinct peaks (Fig. 1EF). The peaks arising in the MBT are important for development. For example, the mature Hb protein pattern (Fig. 1F) has a distinct peak just anterior of mid-embryo (corresponding to the central mRNA peak, Fig. 1CE). If this peak is removed, downstream gene expression in parasegment 4 (PS4) is affected and thoracic segment 2 (T2) does not form properly [33]. Quantitative modelling of earlier, broad patterning has been used to characterize Bcd-Hb regulatory interactions and how these contribute to spatial precision, e.g. [23,34–37]; the aim of the current work is to use quantitative modelling of mid-NC14 pattern refinement to understand how gap-gap interactions in the MBT contribute to spatial precision.

*hb* has extensive cis-regulatory regions with binding sites (BSs) for maternal and gap transcription factors (TFs). *hb* expression varies as TF patterns and the bound state of its cis-regulatory regions vary. *hb* has two promoters with distinct transcripts: the proximal P2 active in NC10-NC14 Bcd-dependent *hb* expression; and a distal P1 promoter with early ubiquitous expression and later ‘striped’ expression in the PS4 and posterior peaks [38,39]. The proximal regulatory region has been extensively studied [39,40]: BSs for Bcd, Hb and the product of the gap gene *Krüppe*l (*Kr*) were found by DNA footprinting [41]; and lacZ reporters driven from this region recapitulate the early, broad anterior ‘step’ pattern of *hb* [42]. Recent work shows that *hb* is under the control of 3 distinct enhancers: the proximal ‘classical’ enhancer; a ‘shadow’ enhancer; and a ‘stripe’ enhancer [18,43]. Expression driven by the classical and shadow enhancers resembles the early anterior step *hb* pattern; the stripe enhancer drives expression at the PS4 position and the posterior peak. The stripe enhancer was found to be largely regulated by gap TFs (specifically Hb, Kr and knirps (kni)), consistent with a role in mid-NC14 gap-dependent pattern refinement ([44,45]; http://www.iephb.nw.ru/hoxpro/hunchback.html).

Quantitative modelling has been used for several decades to reconstruct and understand the regulatory mechanisms which produce *Drosophila* segmentation patterns. Gap gene models featuring maternal activation and simple inhibition between neighbouring gap domains have shown success in producing WT border positions and their temporal shifts (e.g. [26,30]), but have shown less success with mutant phenotypes and fine-scale mid-NC14 pattern features (peaks). Peak formation indicates gene-gene interactions which are more complex than simple inhibition. Here, we model *hb* patterning and peak formation at the mid-embryo, where the sharp drop in anterior *hb* expression is bounded to the posterior by the Kr domain (Fig. 1BDF, green), to characterize such interactions.

Simple inhibition has been measured both in vivo and in vitro for a number of gap genes [46–50]. At the mid-embryo, early studies showed mutual inhibition between *hb* and *Kr* [51], with *hb* shifting posteriorly upon removal of Kr (also observed by [52]), and Kr shifting anteriorly in the absence of Hb (as well as the Kr sites in the *hb* regulatory region [41], Hb also binds in the *Kr* cis-regulatory region [53]).

However, Hb and Kr also show more complex activities as transcriptional regulators. In vitro, it was found that low levels of Hb could be activating while higher levels could be inhibiting [54]. Such dual concentration-dependent action was subsequently observed in the embryo, where it was found that Hb could both activate and inhibit *Kr* [55]. This dual activation-inhibition by Hb was recently incorporated into a quantitative model of Kr expression [56].

Similarly, Kr can act as an activator at low concentration and as an inhibitor at high concentration [57]. Protein-protein interactions (including with Hb) can modulate whether Kr acts as an activating or inhibiting TF [50] (e.g. Kr monomer can act as an activator, while the homodimer can be inhibitory [58]). Recent data suggests both roles are active in the embryo. If Kr BSs are removed from a fragment of the *hb* stripe enhancer (which lacks PS4 expression), reporter expression expands posteriorly [43], indicating Kr inhibition. For intact *hb* cis-regulatory regions, however, *Kr-* mutant embryos show an anterior *hb* shift and loss of the mid-embryo Hb PS4 peak [59]. This indicates that Kr plays an activating role in *hb* expression, particularly at PS4 (potentially through BSs in the complete stripe enhancer). Further data [60] indicates that Kr is the principal gap regulator of *hb* in the mid-embryo (around 35–65 percent egg length, %EL): in this region, *kni-* mutants show lowered levels of Kr, Hb and Giant (Gt), but very little alteration in the shape of their expression profiles (including unaltered Hb PS4); in *Kr-* embryos, while the small posterior Gt peak shifts anteriorly, the large anterior Gt peak adjacent to Hb PS4 is unaltered. Loss of Hb PS4 in *Kr-* embryos is therefore likely to be directly due to loss of Kr activation (rather than via an indirect Gt effect).

Formulating these results into a quantitative model allows us to characterize the regulatory dynamics underlying mid-embryo pattern refinement. Previously, we developed a Bcd-Hb model for the early (unrefined) anterior step pattern of Hb [37]. By building the model from data on reporter constructs of the proximal promoter, a *hb* mutant, and WT, we characterized the relative contributions of Bcd and Hb (self) activation. With stochastic simulations, we computed the propagation of intrinsic expression noise during *hb* activation, finding aspects of the regulation (such as multiple Bcd BSs and self-activation) which attenuated noise and could contribute to the overall robustness of segmentation.

In the current project, by extending the Bcd-Hb model to include Kr (with the complex regulatory dynamics indicated by experimental results), we can begin to characterize how gap-gap interactions refine gap domains in mid-NC14. In particular, our model quantifies a dual regulation of *hb* by Kr to form the Hb PS4 peak. To our knowledge, it is the first mathematical model of the gap ‘striping’ which precedes the striped patterning of the downstream pair-rule and segment polarity genes (for example, Hb PS4 expression is necessary for *fushi-tarazu* stripe 2 expression and *engrailed* stripe 4 expression [33], respectively). We then use the model to compute noise propagation within this regulatory framework to find aspects of gap-gap regulation which can limit expression noise and contribute to developmental robustness. This indicates that Kr regulation could reduce *hb* expression noise in several ways. We predict that *Kr-* mutants should show increased variability of the Hb mid-embryo boundary position; this has now been observed experimentally [60]. We also predict that Kr increases the determinacy of *hb* expression in the boundary region, which may be observable by high resolution imaging of *hb* transcripts.

## Model and Methods

### Background and approach

Our current Bcd-Hb-Kr model is extended from our previous Bcd-Hb model [37] for early NC14 Hb expression. The Bcd-Hb model was developed from data on the ‘classical’ proximal enhancer. It calculated changes in the bound state of the enhancer (how many Bcd and Hb BSs are bound, *b*_*x*_ and *h*_*x*_ respectively) due to TF binding/unbinding at 6 Bcd BSs and 2 Hb BSs, as well as *hb* mRNA transcription (rate dependent on the *b*_*x*_, *h*_*x*_ state of the enhancer) and decay, and Hb protein translation, decay, and diffusion. Differential equations for these rates were solved at nuclear resolution over the length of the AP axis (100 nuclei), for the first 30 minutes of NC14.

The 6 Bcd sites represented the 3 strong affinity and 3 weak affinity sites characterized by footprinting in [40]. BS-binding and transcription rates were set by modelling expression data from a series of reporter constructs made with different combinations of these BSs [42] (all of which were Bcd activated, with anterior-high/posterior-low expression patterns). Increasing binding strength shifted the high-low boundary to the posterior. Starting from a construct with a single BS, we used the experimental boundary positions to sequentially build up and set the relative model binding constants for the 6 BSs. Expression intensity also increased with number of BSs. We used the relative experimental intensities to sequentially (starting from data for 1 BS) set the relative transcription rates in the model for each bound state (*b*_*x*_).

**Fig 2:**
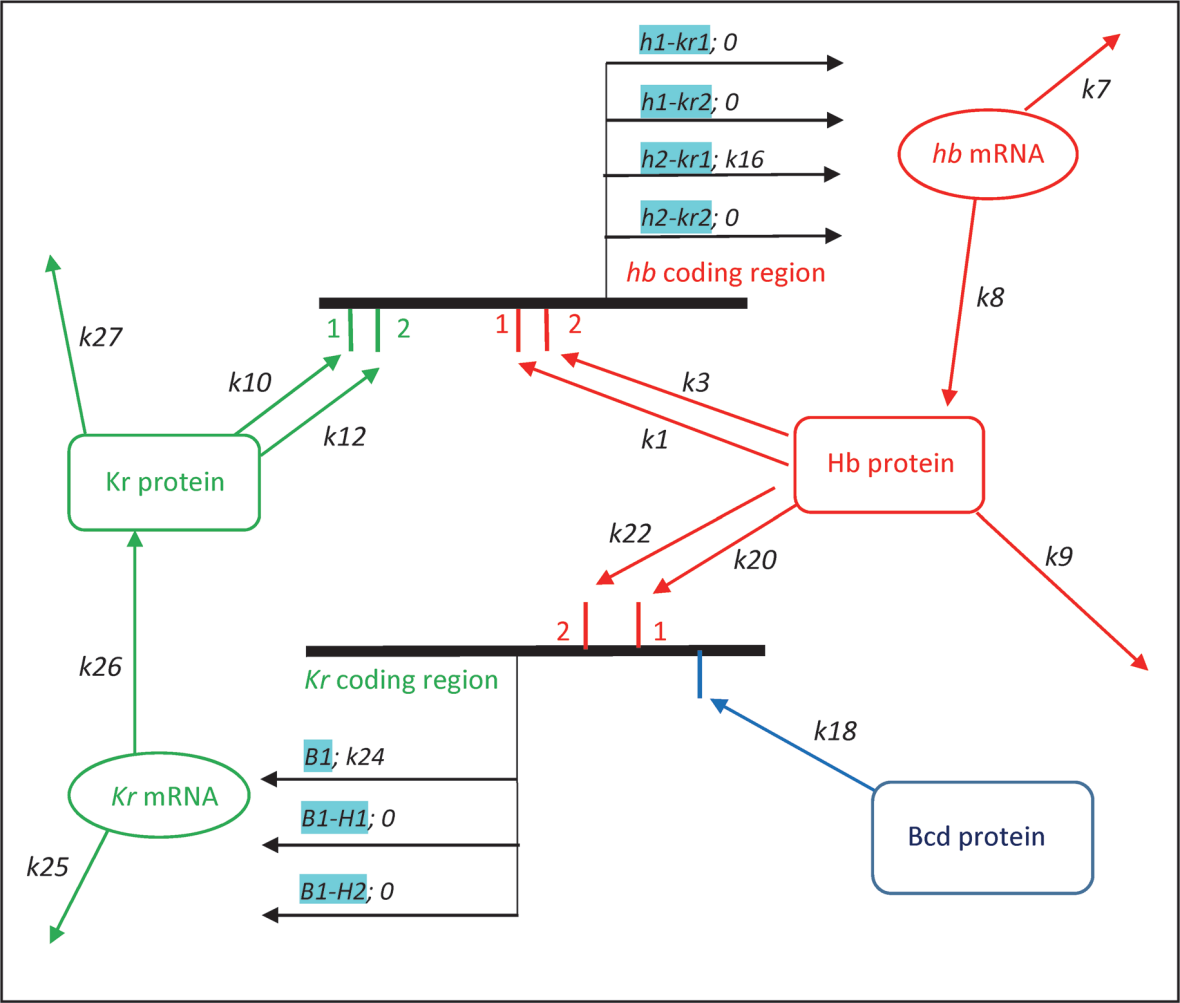
Hb-Kr regulatory model. Schematic of *hb* (top, red) and *Kr* (bottom, green) genes (black bars); regulatory reactions represented by arrows, with rate constants *k*. A complete tabulation of the elementary reactions of the model and the *k* values are given in Tables 1–3. Protein TFs bind regulatory regions for each gene (in reversible reactions); transcription rates depend on the bound state of the TF BSs (cyan; e.g. *h1-kr1* indicates that the *hb* cis-regulatory region has 1 Hb BS bound and 1 Kr BS bound). ***hb***: overall transcription rate consists of Bcd- and Hb-dependent components (Table 1; and Fig. 2 of [37]), and Kr-dependent components (shown, top; note Hb as a co-factor; see also Table 2). Dual regulation—the Kr contribution is highest when a single Kr is bound (*k*_*16*_ term), and goes to 0 when a 2^nd^ Kr is bound. ***Kr***: Bcd protein activates *Kr* transcription (bottom, *k*_*24*_ term); Hb protein inhibits Kr transcription (0 transcription when Hb bound; see also Table 3). (In the **Hb dual** and **dual-dual** variations of the model, *Kr* is activated not by Bcd, but by the 1^st^ Hb binding.) *hb* and *Kr* mRNA are translated, and both mRNA and proteins decay. Spatially, Hb and Kr protein diffuse between nuclei.

The 2 Hb BSs in the model represented the 2 sites found in the proximal enhancer by footprinting [41] (Hb self binding is represented in red, Fig. 2, top, *k*_1_ and *k*_3_ constants; for Bcd BSs, see Fig. 2 of [37]). Binding constants for the Hb sites were determined by the posterior shift (activation) in boundary position from the self-regulation mutant *hb* ^14F^ to WT, and the accompanying increase in expression intensity determined the Hb-dependent transcription constants.

Data (positions and expression intensities) and the corresponding model parameters (binding and transcription constants, respectively) are given in S1 Text and Tables S1–S3 of [37]. With the parameters set in this way, we then solved the model stochastically (i.e. with rate constants defining reaction probabilities; see Computations section below) to characterize the intrinsic noise generated during gene expression and to identify noise-reducing components of the regulatory mechanism.

### Adding Kr

From this BS-level model for the early Bcd-dependent Hb ‘step’ pattern, adding Kr allows us to characterize gap-gap dependent mid-NC14 pattern refinement of Hb, in particular at the mid-embryo (formation of the PS4 peak). As with the Bcd-Hb project, we set the model parameters by matching deterministic simulations (differential equations) to experimental positions and expression intensities, then use stochastic simulations (probabilistic rate equations) to study the effects of gap-gap regulation on intrinsic noise in *hb* expression. Adding Kr involved **a**) adding Kr BSs to the *hb* cis-regulatory region, and **b**) formulating a model for *Kr* expression. Fig. 2 gives a schematic of the Hb-Kr dynamics in the model (*hb* transcription—top, *Kr* transcription—bottom); the complete mechanism and parameters are given in Tables 1–3. For **a**), Kr BSs in *hb* cis-regulatory regions are well characterized—in particular the 2 Kr BSs in the classical *hb* enhancer [41], and more recently Kr sites in other *hb* enhancers [43]. To account for Kr effects on *hb* expression (e.g. [59]), addition of 2 Kr BSs to the *hb* model allows a minimal representation of Kr dual cis-regulation, in which the *kr*_x_ bound states (*x* = 1 or 2) have opposite effects (Fig. 2, top, in green, *k*_10_ and *k*_12_ constants; black arrows represent transcription, rates depend on which BSs are bound, cyan). For **b**), building from the dual regulation model of [56], we added a cis-regulatory region for *Kr* (Fig. 2, bottom) with 2 Hb BSs (*k*_20_ and *k*_22_ constants) and 1 Bcd BSs (*k*_18_ constant; note—there are Bcd BSs for *hb* not shown in Fig. 2, see previous section and [37]), and modelled *Kr* transcription, translation, decay and protein diffusion.

**Table 1:** Hb reactions: Hb TF binding; effect of bound-state on transcription; translation; decay (mRNA and protein).

$h_{0}+H\overset{k_{1}=2.2e6}{\underset{k_{2}}{⇌}}h_{1}$	$h_{1}+kr_{0}\overset{k_{5}=5e9}{\to }MH+h_{1}+kr_{0}$	$MH\overset{k_{7}=6.8e-3}{\to }\mathrm{decay}$
$h_{1}+H\overset{k_{3}=3.4e8}{\underset{k_{4}}{⇌}}h_{2}$	$h_{2}+kr_{0}\overset{k_{6}=4.5e10}{\to }MH+h_{2}+kr_{0}$	$MH\overset{k_{8}=3.125e-1}{\to }H$
		$H\overset{k_{9}=6e-3}{\to }\mathrm{decay}$

*h*_*x*_ represents the number of Hb sites bound; H is Hb protein; *kr*_*x*_ is the number of Kr sites bound; MH is *hb* mRNA.

**Table 2:** Kr effects on *hb* regulation: Kr TF binding; effect of bound state on transcription.

$kr_{0}+\text{Kr}\overset{k_{10}=8e7}{\underset{k_{11}}{⇌}}kr_{1}$	$h_{1}+kr_{1}\overset{k_{14}}{\to }MH+h_{1}+kr_{1}$	$h_{2}+kr_{1}\overset{k_{16}=1.25e11}{\to }MH+h_{2}+kr_{1}$
$kr_{1}+\text{Kr}\overset{k_{12}=1e7}{\underset{k_{13}}{⇌}}kr_{2}$	$h_{1}+kr_{2}\overset{k_{15}}{\to }MH+h_{1}+kr_{2}$	$h_{2}+kr_{2}\overset{k_{17}}{\to }MH+h_{2}+kr_{2}$

Kr is the Kr protein.

**Table 3:** *Kr* regulation: Bcd and Hb TF binding; effect of bound state on transcription; translation; decay (mRNA and protein).

$KrB_{0}+B\overset{k_{18}=1.8e7}{\underset{k_{19}}{⇌}}KrB_{1}$	$KrB_{1}+KrH_{0}\overset{k_{24}=4.5e11}{\to }MKr+KrB_{1}+KrH_{0}$	$MKr\overset{k_{25}=6.8e-3}{\to }\mathrm{decay}$
$KrH_{0}+\text{H}\overset{k_{20}=5e5}{\underset{k_{21}}{⇌}}KrH_{1}$	$KrB_{1}+KrH_{1}\overset{}{\to }\mathrm{no transcription}$	$MKr\overset{k_{26}=3.57e-1}{\to }Kr$
$KrH_{1}+\text{H}\overset{k_{22}=8e9}{\underset{k_{23}}{⇌}}KrH_{2}$	$KrB_{1}+KrH_{2}\overset{}{\to }\mathrm{no transcription}$	$Kr\overset{k_{27}=6e-3}{\to }\mathrm{decay}$

*KrY*_x_ is the bound state of transcription factor Y; B is Bcd protein; MKr is *Kr* mRNA.

### Hb-Kr interactions

Hb-Kr interactions of simple inhibition, simple activation and dual regulation can be modelled within this framework. Past modelling indicates that simple mutual inhibition (**mut inh**) between gene pairs does not produce refined peak patterns (e.g. [16,17,26]). From the experimental evidence for dual regulation (Hb and Kr acting as both activators and inhibitors depending on their concentrations), there are three possibilities to explore: Hb dual acting on *Kr*, Kr simply inhibitory on *hb* (**Hb dual**); Hb and Kr both dual acting on each other (**dual-dual**); and Kr dual acting on *hb*, Hb simply inhibitory on *Kr* (**Kr dual**). **Hb dual** is an implementation of the model of [56], but with dynamic Hb (Hb was static in [56]).

In the **Hb dual** mechanism, Hb produces its own posterior inhibitor, but Hb itself remains an unrefined anterior ‘step’ pattern (see Results). Experimentally, the anterior shift and loss of Hb PS4 expression in *Kr-* mutants [59] indicate that Kr has a positive effect on *hb* expression. Both the **dual-dual** and **Kr dual** mechanisms have this, activating *hb* upon binding of the 1^st^ Kr BS (Fig. 2, *k*_10_ and *k*_16_ parameters), and inhibiting *hb* on binding of the 2^nd^ Kr BS (Fig. 2, *k*_12_ parameter; Kr-dependent transcription off). In molecular terms, the 2^nd^ bound BS could hinder activity at the 1^st^ BS at short range (see [61] on quenching by Kr) or long range; the **Hb dual** ‘dual P’ mechanism proposed in [56] envisions “occupancy of all of the sites leads to the masking of the Hb activation domain”. Also, Hb PS4 peak formation on the anterior edge of the Kr domain (and not the posterior) indicates that Hb is a co-factor, and therefore that Kr activation occurs at high Hb concentration (*h*_2_ state; Fig. 2, top, cyan). We found that the **dual-dual** mechanism tends to form a Hb domain which moves continuously to the posterior (see Results). This movement can be stabilized with the **Kr dual** mechanism, in which *Kr* is activated by the steady-state Bcd profile (*k*_18_ parameter), rather than with the dynamic Hb. **Kr dual** (dual activation-inhibition by Kr of *hb*, simple inhibition of *Kr* by Hb) therefore provides the best match to the data and is used for the main conclusions of this paper.

### Parameters

We used deterministic simulations of the **Kr dual** model to find parameters (*k* values, Fig. 2) matching experimental data on positions (binding constants) and expression intensities (transcription rate constants). Bcd-dependent *hb* expression parameters were unaltered from [37]. New data was used to set the Kr-dependent parameters, and to adjust the Hb parameters.

### Hb self-regulation

The Hb-dependent events in *hb* expression are shown in Table 1 (see also Fig. 2). Binding is modelled as the reaction between a Hb TF (*H*) and a Hb BS (*h*_*x*_). For the Bcd-Hb model in [37] (without Kr), these binding constants were set by matching the WT Hb boundary position of 47%EL (30 minutes into NC14, data from [23]). Since we are now modelling Kr regulation of more mature pattern, the Hb binding constants were reduced to match the Hb boundary position of 44%EL reported for *Kr-* [59], 40 minutes into NC14: *k*_1_ = 2.2e6; *k*_3_ = 3.4e8 (M^−1^s^−1^, see Computations section below regarding units). (Positive self-effects were modelled as in [37] (see also [39]); negative self-effects [43,19] would not alter the conclusions regarding Kr regulation.) All unbinding rates in the model (e.g *k*_2_, *k*_4_) were set to unity, therefore the binding rates are relative. It was reported in [59] that *Kr-* expression intensity is half that of WT. Therefore, transcription rates dependent on Hb occupancy (*h*_1_ or *h*_2_ states), in the absence of Kr (*kr*_0_ state), were also reduced from [37]: *k*_5_ = 5e9, *k*_6_ = 4.5e10.

### Concentrations

Due to the inverse relationship of noise with concentration (in general, the higher the concentration the lower the relative noise), concentration levels are highly pertinent in stochastic modelling. Correspondingly, qualitatively different noise behaviour in experiments can be used to refine estimates of in vivo concentration levels. Concentration estimates in the segmentation system were initially made for Bcd, ranging from maximal Bcd protein of around 1000 molecules/nucleus [62], to approximately 7000 molecules per nucleus (with surrounding cytoplasm) [63], to about 20,000 molecules/nucleus [64]. Work in [62] indicated that Bcd and Hb have similar protein concentrations. Recent mRNA results provide a more direct quantitation of *hb* levels. Boettiger reported anterior concentrations of 300–500 copies per nucleus (2011 *Drosophila* Conference plenary). This was recently reported [19] at about 300 copies per nucleus and surrounding cytoplasm (cylinders of 12μm depth *x* 5μm diameter) in NC13 (corroborated in live embryos [20]); up to 600 copies in early NC14; and dropping to 300–400 copies anterior of PS4 in mid-NC14. Another recent single molecule FISH study indicated NC13 levels of 200–300 *hb* mRNA per nucleus+cytoplasm (for all depths, ∼100/nucleus in the nuclear layer; Heng Xu and Ido Golding, personal communication). This is similar to the ∼100 mRNA copies per cell found for HOX genes later in segmentation [65].

Computational initial conditions, for *t* = 0 at the onset of NC14, correspond to the experimental values for the end of NC13. For the nuclear layer (computational units of (5μm)^3^ represent a nucleus plus neighbouring cytoplasm), initial maximal *hb* mRNA was ∼100 copies per nucleus (+ cytoplasm). This rose to ∼140 copies by mid-NC14. A fairly typical translational efficiency of 1:50 (mRNA:protein; *k*_8_ = 3.125e-1 (s^−1^); e.g. [66]) produced mid-NC14 Hb protein levels of 7000 per nucleus (comparable to Bcd-GFP measurements [63]). Computations at these concentrations are corroborated by experiments (see Results); we also explored noise output at a range of other concentration levels (see Discussion).

### Timescale

Initial patterns for *hb* and *Kr* mRNA and protein (*t* = 0), based on NC13 FlyEx data (urchin.spbcas.ru/flyex), were 60% of mid-NC14 values. The increase to mature mid-NC14 Hb levels in 40 minutes reflects the production and decay rates—overly fast rates reach mature levels too soon, overly slow rates do not give the observed increase within NC14. The production rates in the sections above and below, with decay rates of *k*_7_ = 6.8e-3 for *hb* mRNA and *k*_9_ = 6e-3 for Hb protein, amplify Hb the observed amount on the correct timescale.

### Hb-Kr interactions

#### Kr regulation of hb

Kr dual action on *hb* (**dual-dual** and **Kr dual** mechanisms) is implemented through the events shown in Table 2, with successive binding of two Kr TFs changing the BS state (*kr*_0_→*kr*_1_→*kr*_2_).

**Fig 3:**
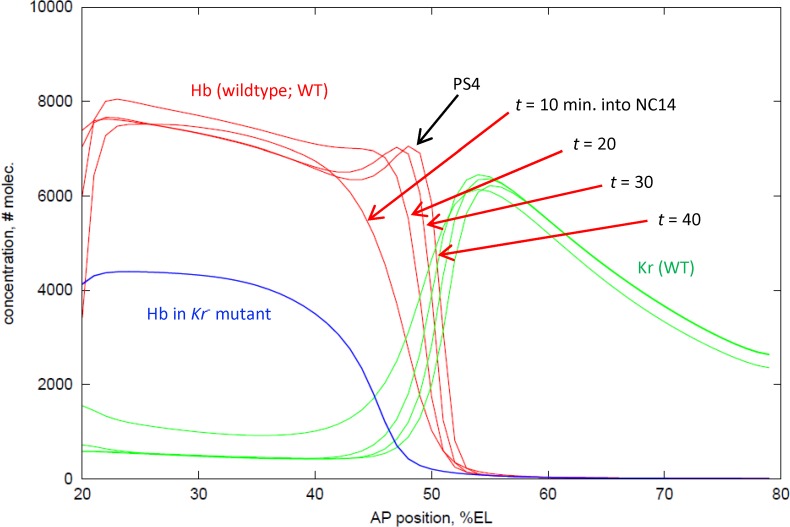
Hb PS4 formation by Kr dual regulation (activation/inhibition). Red curves: Hb protein concentration profiles along the AP axis, at 10, 20, 30, 40 minutes into NC14. Profiles at 10–20 minutes show the early ‘step function’ expression observed experimentally; later profiles show the development of the PS4 stripe, on the correct timescale. Green curves: Kr protein concentration profiles, at the same times. Blue curve: Hb expression (at 40 minutes) in *Kr-*. See Fig. 1BDF for comparison to experimental data over this period.

The relatively strong binding of the 1^st^ Kr, *k*_10_ = 8e7, places the *kr*_1_ state at low Kr concentration, such that its activation of *hb* transcription (*k*_16_ = 1.25e11) occurs at the anterior edge of the Kr peak, in the PS4 position. Binding strength (*k*_10_) was set by positioning the Hb PS4 peak (48%EL at *t* = 40 mins.; see Fig. 3, Results,) relative to the Kr peak (55%EL at *t* = 40 mins.; Fig. 3). (Early NC14 positions match data, Fig. 1, later Hb is about 5%EL posterior of data; Kr dual regulation, in addition to activating expression at PS4, can introduce a posterior bias.) Transcription (*k*_16_) is set by the increase (doubling) of Hb expression from *Kr-* mutants to WT [59]. Hb co-action (reflected by Hb peak formation only to the anterior of Kr) is incorporated by high transcription (*k*_16_) for the *h*_2_-*kr*_1_ bound state and zero transcription (*k*_14_) in the *h*_1_-*kr*_1_ state. (*h*_1_ effects are not strong: in tests with *k*_14_ = *k*_5_ (the *h*_1_-*kr*_0_ rate) positions were unchanged; testing *k*_*14*_ = *k*_*16*_ (the *h*_2_-*kr*_1_ rate) shifted Hb expression 1%EL to the posterior.)

The 2^nd^ Kr has a lower binding constant, *k*_12_ = 1e7, so the *kr*_2_ state occurs at higher Kr concentration, to the posterior of PS4. (The weaker binding of *kr*_2_ than *kr*_1_ could represent steric hindrance, rather than innately different BS affinities.) Gap-regulated *hb* transcription is zero in the *kr*_2_ state (*k*_15_ and *k*_17_ parameters are zero; the *kr*_*x*_ state does not affect basal Bcd activation of *hb*). *kr*_2_ inhibition limits the posterior extent of Hb: *k*_12_ is set to give a half-height Hb boundary position (51%EL) intermediate between the PS4 peak and the Kr peak (cf. Fig. 1F).

#### Regulation of Kr transcription

*Kr* expression dynamics are modelled by the events shown in Table 3. *KrB*_*x*_ and *KrH*_*x*_ represent the bound state of the BSs in the *Kr* cis-regulatory region, for Bcd and Hb TFs, respectively. In tests with the **dual-dual** (cf. [56]) mechanism, *Kr* transcription was activated in the *KrH*_*1*_ state and inhibited in the *KrH*_*2*_ state, with no effect from *KrB*_*1*_. Due to the excessive posterior shifting with **dual-dual**, we implemented the **Kr dual** mechanism for *hb* expression, with parameters as shown in Table 3. Here, the *KrB*_*1*_ state activates *Kr* transcription (at rate constant *k*_24_ = 4.5e11) and the *KrH*_*1*_, *KrH*_*2*_ states are inhibitory (have zero transcription). The values of the Bcd and Hb binding constants (*k*_*18*_ = 1.8e7, *k*_20_ = 5e5 and *k*_22_ = 8e9, respectively) control the Kr peak position (55%EL). *Kr* mRNA and protein decay constants (*k*_25_ and *k*_27_, respectively) are set equal to *hb*’s (*k*_7_ and *k*_9_). The Kr translation rate constant, *k*_26_ = 3.57e-1, was adjusted to match FlyEx data (at timeclass 6, T6, 40 minutes into NC14) on Kr peak height relative to Hb.

### Diffusivities

Patterning in the model was primarily local (see also [67]): mRNA diffusion was not modelled; interaction between nuclei was via protein diffusion. A Hb diffusivity, D_Hb_, of 3e-10 cm^2^/s gave a sharp Hb boundary and limited posterior shifting; a faster Kr diffusivity, D_Kr_ = 3e-9 cm^2^/s, gave the observed breadth of the PS4 peak (slower D_Kr_ gave a sharper PS4 peak).

### Computations

Stochastic and deterministic solutions of the model were computed with the MesoRD software package ([68]; http://mesord.sourceforge.net). 1D computations were solved in a one-dimensional series of 60 subvolumes (each a cube 5μm on a side), corresponding to energids (nucleus plus cytoplasmic neighbourhood) along the AP axis (between 20 and 80%EL). 2D computations were run on a rectangular grid of 600 subvolumes (60AP by 10DV (dorso-ventral)). Computations solved for model species densities in each subvolume, according to the specified reactions (Tables 1–3) and between-subvolume protein diffusion. Deterministic simulations used a 4^th^ order Runge-Kutta solution method. For stochastic solutions, MesoRD solves the reaction-diffusion master equation, in which each reaction and diffusion event has a probability (set by the macroscopic rates) of occurring in a unit of time. The software implements the next subvolume queuing method [69,70] to significantly improve memory and processing requirements, making computation possible for the number of species and subvolumes in the *hb-Kr* model.

Computations are run in real units (μm, s, etc.). However, 2^nd^ order rate constants are elevated due to the nuclear resolution of the model. Since a BS state (e.g. *h*_2_) is modelled as a chemical species (molecule), transcription for bound-states with different TFs, such as *h*_2_-*kr*_1_, is 2^nd^ order, as are binding events. These rate constants are in units of M^−1^ s^−1^, varying with concentration. Single BS state ‘molecules’ in the computed (5μm)^3^ nuclear volume represent very low concentration. 2^nd^ order rate constants are correspondingly high for overall reaction rates (= concentrations * rate constant) to match the observed large-scale NC14 expression dynamics (e.g. the near doubling of Hb level in half an hour). Smaller computational units could provide closer estimates of absolute rate constants, but would be computationally intensive and would not contribute to the aim of understanding noise effects on embryo-scale patterns at the nuclear resolution of the data. The model therefore has predictive value for the relative rates in the regulatory mechanism, but not for the absolute values of the rate constants.

## Results

As described above, the model was developed and parameters were selected by matching deterministic model solutions to positions and relative intensities in mid-NC14. In particular, the difference between *Kr-* and WT Hb PS4 features constrains potential Hb-Kr dynamics and indicates a role for dual regulation in Hb pattern refinement.

With the parameters set by deterministic modelling, stochastic simulations were used to investigate how Hb-Kr interactions can affect noise propagation during the pattern refinement (MBT) stages of segmentation. We tested the ability of Hb-Kr interactions to reduce noisy expression along their interface, within individual embryos; to reduce between-embryo variability of the Hb boundary position; and to reduce Hb expression variability between transcription centres within nuclei.

### Kr dual regulation can form the Hb PS4 peak: deterministic results

Previous *hb* models have produced the non-refined anterior *hb* step pattern, either through Bcd-Hb dynamics (e.g. [36, 37]) or through single-role gap-gap interactions (primarily inhibitory, e.g. [26]). Within the current modelling framework, we confirmed that simple inhibition of *hb* by Kr creates simple Hb step pattern, using test scenarios (S1 Fig.) with static Kr inhibiting *hb*, with mutual Hb-Kr inhibition (**mut inh**), and with the **Hb dual** mechanism (see Methods).

Adding Kr activation, reflecting the increase in Hb intensity and posterior shift going from *Kr-* mutants to WT [59], produced the refined Hb PS4 peak, both with the **dual-dual** and the **Kr dual** mechanisms (see Methods). Due to the posterior shifting in **dual-dual** (S2 Fig.), **Kr dual** is used to model WT expression (Fig. 3). Binding of the 1^st^ Kr is relatively strong (*k*_*10*_ binding constant, Fig. 2), such that the *hb*-activating *kr*_*1*_ state occurs at the ‘foot’ of the Kr peak, forming Hb PS4 in this position. Binding of the 2^nd^ Kr is weaker (*k*_12_ = *k*_10_/8; Fig. 2), making the *hb*-inhibiting *kr*_2_ state predominant at the Kr peak. In effect, Kr regulates both the anterior and posterior sides of the Hb mid-embryo boundary. Fig. 3 shows computational results from early-through mid-NC14 (red curves, Hb; green curves, Kr; at *t* = 10, 20, 30, and 40 minutes). Computations start from experimental profiles for Hb and Kr at *t* = 0. The computational time series recapitulates the experimentally observed transition from early step pattern (Fig. 3, *t* = 10; cf. Fig. 1B; vertical scale, note experimental results shown in relative intensity [0,1], computations shown in numbers of molecules) through intermediate stages (Fig. 3, *t* = 20; cf. Fig. 1D, with the beginnings of the PS4 ‘shoulder’) to later peaked pattern (Fig. 3, *t* = 30, 40; cf. Fig. 1F). *t* = 10, 20 computations correspond to experimental Hb half-height and Kr peak positions. By *t* = 40, the model reproduces (with a Hb posterior shift, see Methods) the positions and relative heights of the Kr and Hb PS4 peaks, the Hb posterior boundary, and the Hb trough anterior of PS4 (89% of PS4 peak height, as reported in FlyEx at T6). Simulation of *Kr-* mutants, by removing binding of Kr in *hb* (*k*_*10*_ = *k*_*12*_ = 0), produces the loss of Hb PS4, loss of boundary sharpness, and anterior shift (blue curve, Fig. 3) reported in [59]. From the Bcd-Hb ‘baseline’ production of the *Kr*-mutant pattern, WT Hb PS4 is formed by sufficient *kr*_1_ activation to generate the peak, coupled with the correct level of *kr*_2_ inhibition to position the Hb boundary. (Too weak *kr*_2_ binding, and activation of *hb* engulfs the Kr peak; too strong *kr*_2_ binding, and Hb PS4 is lost).

### Kr regulation of Hb precision: stochastic results

Deterministic solutions of the model (e.g. Fig. 3), represent average outcomes for expression patterns. Stochastic solutions can generate the expected range of outcomes due to intrinsic noise in the gene expression process—i.e. due to the inherent randomness of TF binding, transcription, translation and transport. Each step in the regulatory process can make a unique contribution to the noise signature of a gene’s expression. Stochastic modelling (at the master equation level) can generate the noise distribution intrinsic to the kinetics, rather than imposing assumptions on the type or magnitude of the noise. Model results can predict noise effects for particular experimental perturbations (e.g. mutations). We used this approach with the Bcd-Hb model [37] to determine the roles that multiple Bcd BSs and Hb self-regulation have in controlling *hb* expression noise. Here, we solve the Hb-Kr model stochastically to characterize what aspects of Kr regulation may help make *hb* expression robust to intrinsic noise.

### Hb-Kr interactions limit noise at their interface, within embryos

**Fig 4:**
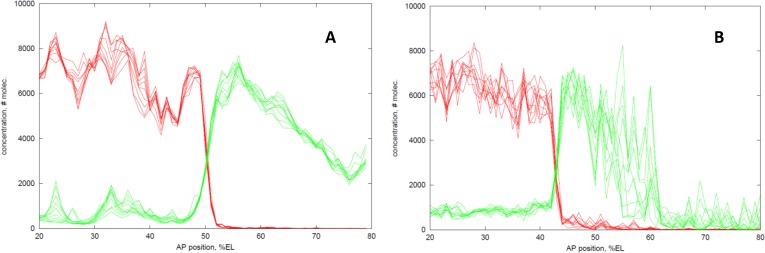
Hb-Kr interactions limit noise at their interface. (A) Stochastic solution of the **Kr dual** PS4 model. Overlay of results at 1 minute intervals over 10 minutes (30–40 minutes into NC14; cf. *t* = 30, *t* = 40 of deterministic solution in Fig. 3). Substantial fluctuations are evident on this timescale in the activated domains, but the mid-embryo interface exhibits much lower noise. (B) The ‘reverse’ **Hb dual** mechanism exhibits comparable noise reduction (but doesn’t form PS4). Same time intervals as (A). Higher overall noise is tested in (B), but the interface remains precise.

Fig. 4A shows a stochastic solution of the **Kr dual** PS4 model, with the same parameters as Fig. 3. Noise levels are determined by the parameters, and are therefore constrained by matching data on positions, expression levels, and timescales. Output is shown at 1 min. intervals, to display the dynamics of the noise at mid-NC14. While Hb and Kr expression are noisy in the non-interface regions, they are much more determinate and temporally stable at their interface: the Hb and Kr boundaries are precise and monotonic (not jagged), with position mapping closely to concentration.

Such interface noise reduction appears to be a general feature of mutual Hb-Kr interaction. Fig. 4B shows a similarly determinate boundary in a stochastic simulation of the ‘reversed’ **Hb dual** mechanism. A mechanism of simple mutual inhibition (**mut inh**) between Hb and Kr (with no cross-activation) also shows significantly lower interface variability (S3 Fig.) than simulations with no Kr (i.e. Bcd-Hb dependent expression). Simulations without mutual feedback, in which a static Kr gradient inhibits *hb*, have interface variability comparable to simulations with no Kr (S3 Fig.). This indicates that mutual Hb-Kr inhibition is an aspect of the interface noise reduction seen with the **Kr dual** PS4-forming mechanism.

Hb-Kr noise reduction is robust to high noise levels. The parameters (and hence noise levels) for the **Kr dual** simulation in Fig. 4A are constrained to fit the NC14 pattern formation timescale. **Hb dual** (Fig. 4B) and **mut inh** (S3 Fig.) have comparable interface variability (to **Kr dual**), despite being run as tests with ten-fold higher protein production and decay rates (producing higher expression noise levels). With Fig. 4A protein rates set as the slowest that will give the Hb rise between *t* = 0 and *t* = 40, it likely represents a conservative estimate of expression noise.

In the **Kr dual** mechanism, Kr activation of *hb* could potentially cause Kr fluctuations to amplify *hb* noise. However, the comparable interface variability between **Kr dual**, **Hb dual** and **mut inh** suggests that if there is such an effect, it is compensated by the noise-reduction of Hb-Kr inhibition.

### Kr reduces between-embryo Hb positional variability

**Fig 5:**
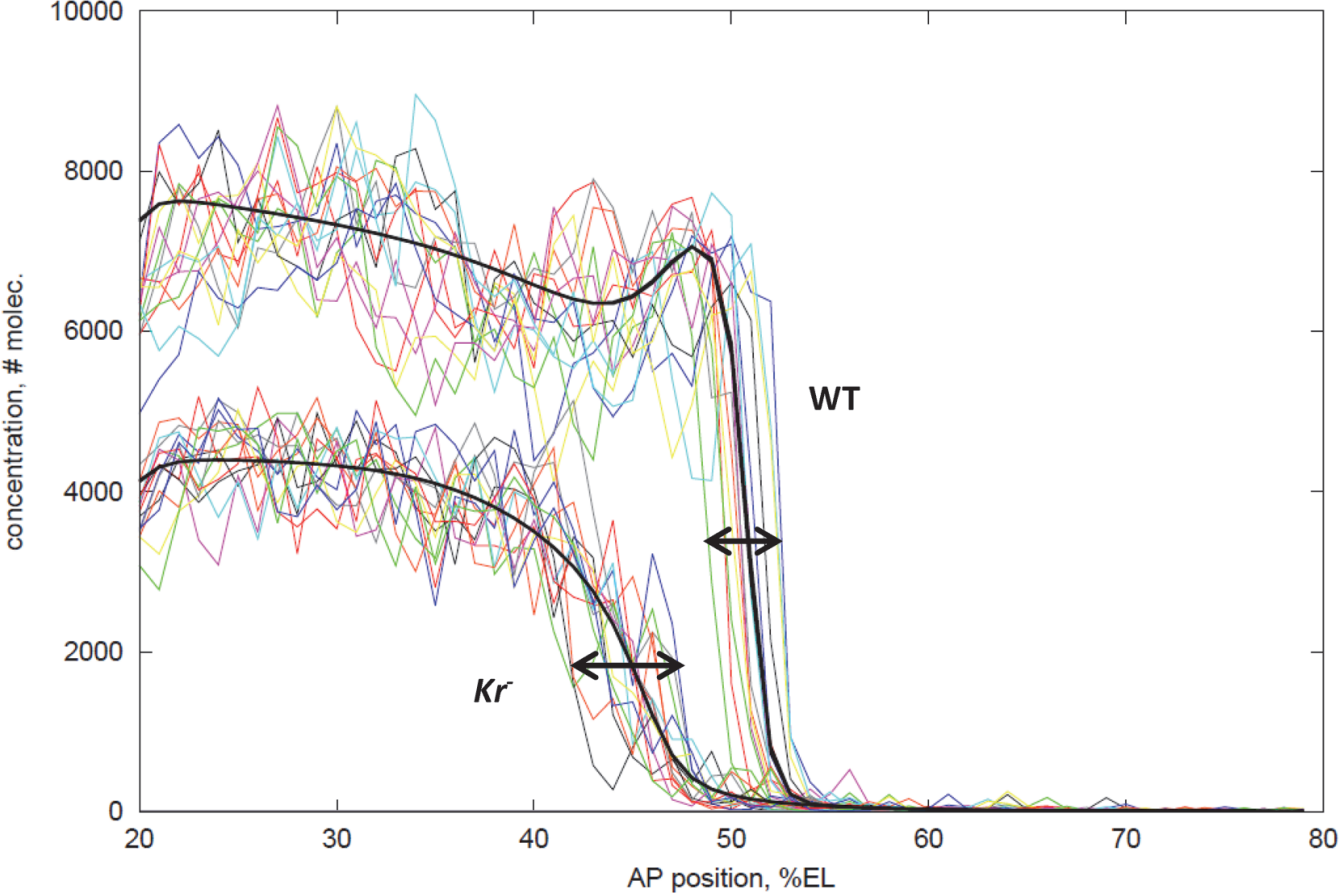
Effect of intrinsic noise on between-embryo variability. Top curves: 25 stochastic simulations of WT (**Kr dual** PS4 model), thick black line is the deterministic result for comparison (cf. Fig. 3). Bottom curves: 25 stochastic simulations of *Kr-*, thick black line—deterministic solution. Arrows indicate the range of positions at which profiles cross half-height. WT has a significantly lower standard deviation in this position than *Kr-*. *t* = 40 minutes into NC14.

The simulations above (Fig. 4) indicate the expression fluctuations which could be expected within an embryo over time. Measurements of such time series would involve live imaging of Hb and Kr protein, for which the technology does not yet exist (though recent progress on live imaging of *hb* mRNA [20, 21] is promising). An approach for measuring noise effects with current fixed embryo staining is to analyze between-embryo variability. Fig. 5 is a computational prediction of what could be observed comparing fixed data (at a *t* = 40 ‘snapshot’) between multiple embryos. In it, 25 WT (**Kr dual** PS4-forming mechanism) and 25 *Kr-* simulations have been overlaid (representing 50 embryos in total). Note that, even in a snapshot, all WT boundaries are monotonic, while *Kr*-boundaries are jagged and non-monotonic. For the boundary position (at half-height, or anterior-most such point for non-monotonic profiles), WT simulations have significantly lower positional standard deviation than *Kr-* simulations (F-test, *p* = 0.012 at *t* = 40 mins.; Table 4). This indicates that Kr’s effect on reducing *hb* intrinsic noise could be observed with between-embryo measurements of Hb positional variability. Recently, experiments [60] have supported these model predictions, finding significantly lower positional standard deviation in WT than in *Kr-* mutants in mid- to late-NC14 (Table 4).

**Table 4:** Positional precision of the Hb mid-embryo border.

	**Model**		**Experiment**	
	**WT**	***Kr-***	**WT**	***Kr-***
**t = 7 min (T1/T2)**	1.0	1.5	1.8	2.0
**t = 20 min (T3/T4)**	1.0	2.1	1.7	1.7
**t = 40 min (T7/T8)**	1.0	1.6	1.0	2.4

Standard deviation of Hb border position at 3 stages of NC14, WT vs. *Kr-*. T indicates timeclasses as used in [60]. Model results are for *n* = 25 simulations each for WT and for *Kr-*. Experimental results are from Table 2 of [60].

### Kr can reduce within-nucleus variability of *hb* expression

In addition to the between-embryo approach discussed above (Fig. 5), newer experimental methods may allow for a more direct measurement of within-embryo expression variability. A number of studies have now reported *hb* expression at ‘nuclear dot’ resolution, visualizing transcription from the different copies of the gene within the nucleus [14, 15, 22,71]. Depending on overall activity, nuclei show 0, 1, or 2 active transcription centres. These reveal temporal integration and synchronization in *hb* patterning prior to cellularization. Recently, it has been shown that *hb* noise scales with the number of active transcription loci, indicating that noise is chiefly intrinsic and independent between nuclear dots [19]. Simulating two independent transcription loci per nucleus (0, 1, or 2 of which can be strongly active), we can predict the dot-to-dot differences arising from such intrinsic noise, and investigate how this is affected by regulation. With the previous Bcd-Hb model, we took such an approach to predict the effect of *hb* self-regulation on within-nucleus variability [37].

**Fig 6:**
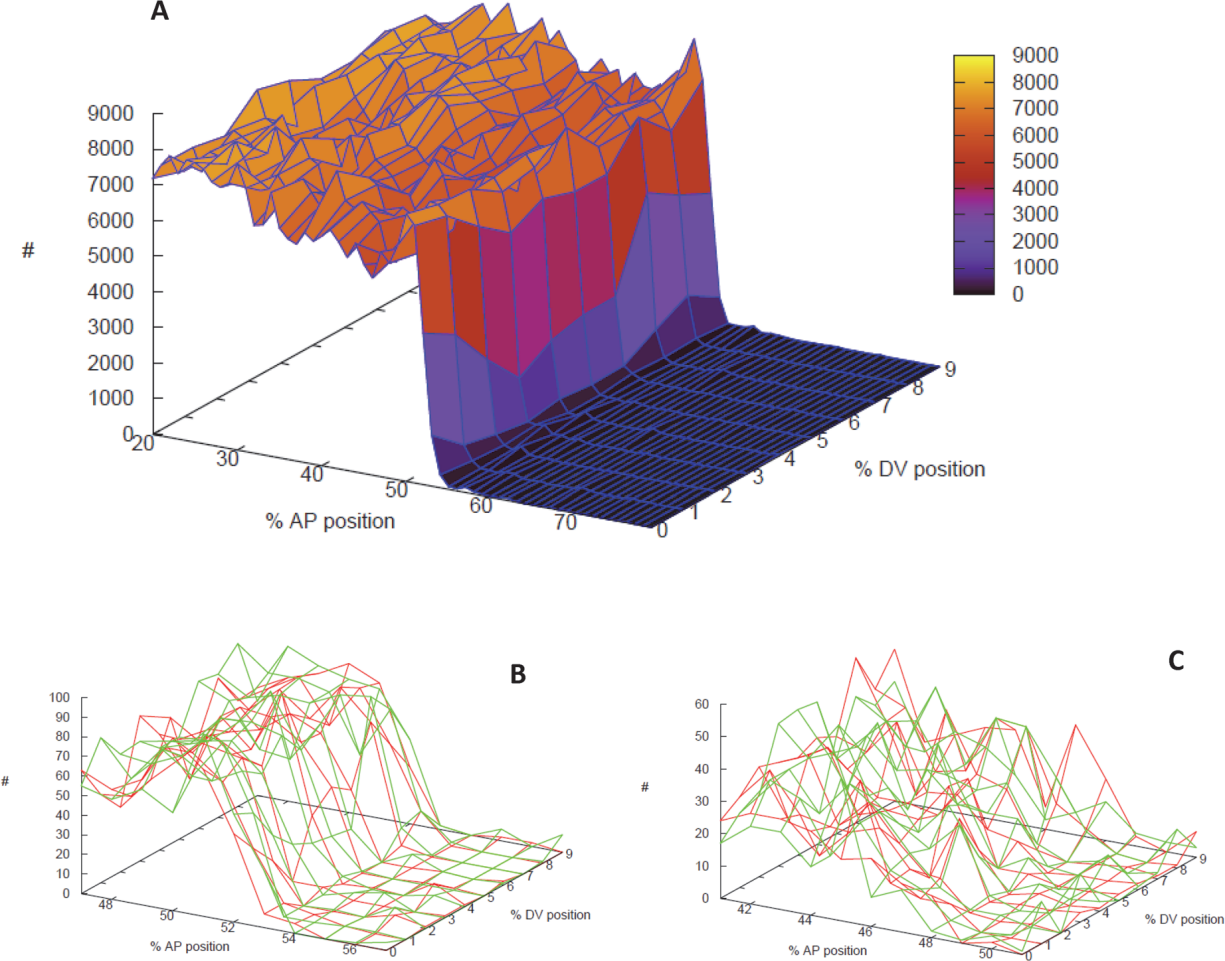
Kr reduction of within-nucleus noise. Stochastic simulations of the **Kr dual** PS4 model with 2 transcription centres per nucleus. (A) WT Hb protein expression surface (note: DV shown stretched relative to AP; actual computational subunits have equal AP, DV dimensions). Vertical axis (and colour scale), number of molecules. (B, C) within-nucleus variability, ±5%EL from the Hb boundary: red and green are *hb* mRNA levels (number of molecules) produced from each of 2 transcription centres per nucleus. Noise is calculated from the relative differences between the red and green levels at each position (Eq. 1). (B) WT; (C) *Kr-*. WT has relatively sharper slope than *Kr-*, and the average within-nucleus noise is significantly lower in WT than in *Kr-*. The simulations shown have average noise levels: 35% for WT (B); 51% for *Kr-* (C). *t* = 40 minutes into NC14.

Here, we have run two-locus stochastic simulations of the **Kr dual** PS4 model to investigate the effects of Kr on within-nucleus *hb* noise and make predictions for experimental observations of nuclear dot differences between WT and *Kr*-. For each nucleus, TF binding and transcription was calculated at loci A and B, with translation creating pooled Hb and Kr proteins. Within-nucleus transcript noise was calculated as a standard deviation of the relative A,B differences in *hb* mRNA:

$${\mathrm{noise}}_{\mathrm{in-nuc}}=\sqrt{\frac{{\sum \left[\left(A-B\right)/\left(\left(A+B\right)/2\right)\right]}^{2}}{m-1}}$$ (1)

where A is *hb* mRNA transcribed at locus A, and B is *hb* mRNA transcribed at locus B. Eq. (1) is calculated over the *m* nuclei with non-zero A and B (corresponding to nuclei with 2 measurable ‘dots’) in the region ±5%EL from the (half-height) Hb boundary. For comparison with 2D image data and larger sample sizes (*m*), simulations were run in 2 spatial dimensions: 20–80%EL in AP; 10% extent in DV (dorsoventral)—i.e., *noise*_*in-nuc*_ was calculated from 100 positions (nuclei) per simulation. Fig. 6A shows the Hb protein expression surface for a simulation on the 2D domain.

2D two-locus simulations were run for the **Kr dual** WT model and for *Kr*-(*n* = 12 simulations each). All WT simulations had lower *noise*_*in-nuc*_ than any *Kr*-simulation. Average *noise*_*in-nuc*_ for WT, 35%, was lower than average *noise*_*in-nuc*_ for *Kr-*, 51%; *p* = 2e-7 (*t*-test). Fig. 6B shows the expression surfaces for the A, B *hb* mRNA copies in a WT simulation with average *noise*_*in-nuc*_ (35%); Fig. 6C shows the expression surfaces for a *Kr-* simulation with average *noise*_*in-nuc*_ (51%). Increase in between-copy scatter, loss of determinacy and loss of slope are apparent in *Kr-* compared to WT. We predict that high resolution *hb* mRNA imaging could distinguish different levels of within-nucleus noise in the mid-embryo: WT embryos should show lower within-nucleus noise than *Kr-* mutant embryos (perhaps observable as a lower synchronization of transcription state in mutants than WT).

## Discussion

In this study, we have developed and tested a quantitative model of mutual interaction between Hb and Kr for regulation of *hb* gene expression at the mid-embryo. Prior models of AP pattern formation in *Drosophila* segmentation have been successful in generating the early broad gap gene expression domains (e.g. [26,30,37]). The present study is, to our knowledge, the first to model the dynamics giving the refined ‘striped’ peak patterns characteristic of the critical mid-NC14 MBT stage of segmentation.

### Dual regulation, PS4 formation

While Bcd activation, Hb self-activation, and simple Kr inhibition can contribute to the non-refined Hb ‘step’ pattern, they are insufficient mechanisms for the refined Hb PS4 peak. Additional regulatory dynamics are needed. By incorporating the dual regulatory (activating and inhibiting) capacity of Kr (measured in [50,57,58]), we modelled formation of the Hb PS4 peak (Fig. 3, **Kr dual** mechanism), and accounted for the loss of PS4 in *Kr-* mutants [59]. These Kr dynamics regulate Hb mid-embryo expression both from the anterior (PS4 activation) and the posterior (inhibition from the Kr peak controlling the extent of the Hb domain).

In addition to observations of WT—*Kr-* differences in mid-NC14, the increasing role for Kr regulation of mid-embryo *hb* in the MBT is supported by recent observations that *Kr* transcription is active in NC14 (just posterior of the PS4 position), well beyond the active phase of anterior Bcd-induced *hb* transcription [22]. The developmental functionality of the Hb PS4 peak, inducing specific locales of downstream pair-rule (*fushi-tarazu* stripe 2) and segment polarity (*engrailed* stripe 4) gene expression [33], indicates that Kr dual regulation of *hb* is a critical element in the formation of the 2^nd^ thoracic segment.

To the extent that the other gap genes are forming peaks in the MBT which affect particular segments, dual regulation may feature more broadly in this critical phase of development. In addition to the work on Hb dual regulation of *Kr* [56] (also [72]), modelling suggests dual regulation by Bcd of *even-skipped* [72]; and, more generally, allowing TF’s to act as either activators or inhibitors for different gene targets improves fits in larger segmentation network models [73].

### Hb-Kr noise reduction

Stochastic modelling indicates that Kr regulation can reduce *hb* expression noise at several levels. To our knowledge, this is the first quantitative characterization of how gap-gap interactions affect intrinsic expression noise.

### Within-embryo, interface noise

First, Kr regulation can make the Hb boundary more determinate (monotonic and non-jagged, with low positional variability in time; Fig. 4). This may be due in part to the mutual Hb-Kr inhibition within the **Kr dual** PS4 model: simulations with simple mutual inhibition (**mut inh**) showed significantly lower boundary noise than simulations with no Kr (but neither of these mechanisms produced PS4). Also see [74] on noise reduction for two mutually-inhibiting gradients. Dual regulation may offer additional precision: recent experimental and theoretical work in yeast has found activation and repression by the same TF can reduce noise in target gene expression [75]. Finally, the capacity of activator-inhibitor dynamics with different diffusivities to reduce noise has been studied for some time [76,77]. Precise expression of *hb* through mutual activation-inhibition kinetics may be part of a broader motif for reliable spatial patterning.

### Between-embryo variability

Second, between-simulation statistics indicate the degree to which *hb* intrinsic noise can be manifested as between-embryo variability in the Hb boundary position, and indicate that Kr regulation plays a significant role in making the boundary precise (Fig. 5). This has now been observed experimentally, with a significantly higher precision between WT embryos than between *Kr-* mutant embryos in mid- to late-NC14 [60]. This is consistent with the suggestion that increased variability in thoracic outcomes upon removal of the stripe enhancer (partially under Kr control) is due to increased variability in the Hb boundary [43]. Experimental observations and the computational results suggest Kr’s role in limiting *hb* intrinsic noise could be a significant factor in limiting between-embryo positional variability.

Chen et al. [78] recently made reporter constructs with Runt (Run) BSs inserted into the proximal *hb* enhancer. Run acts as a repressive posterior-to-anterior gradient in early NC14, and is not significantly patterned by Hb at this stage. Increasing the number of Run BSs in the construct produced increasingly anterior shifts of the reporter expression boundary, but did not show a trend in the boundary’s standard deviation. This suggests that unidirectional repression (Run→*hb*) is not sufficient to reduce positional variability, and that Kr’s reduction of Hb variability depends on their mutual interaction.

The observed noise difference between WT and *Kr-* mutants gives an indication of the protein concentrations involved. Simulations produce the observed difference for anterior Hb protein levels of ∼7000/nucleus, with *hb* mRNA from ∼140/nucleus (Figs. 3–6 results) to tests with ∼700/nucleus (higher than the highest levels measured in [19]). However, in simulations with higher Hb protein concentrations (∼20,000/nucleus, with proportionately higher Kr, and *hb* mRNA at ∼400/nucleus), WT and *Kr-* variability were lower and not as clearly separated. This suggests that Hb and Kr, and perhaps other *Drosophila* segmentation TFs, operate at moderate concentrations (∼7000/nucleus; the same level measured for Bcd in [63]), making them susceptible to intrinsic noise effects. Our simulations indicate that Hb-Kr interactions damp this noise to increase reliability of the Hb border position. Faster production and decay rates, which could produce higher noise and allow for higher concentrations to give the observed WT—*Kr*-noise difference, are not expected, due to the time it takes for Hb pattern to mature in NC14. If rates were slower than estimated in Tables 1 and 2, the experimental WT—*Kr-* noise difference would imply correspondingly lower concentrations. Given the Table 1, 2 estimates of the rates, significantly lower concentrations (than 7000/nucleus) would have an associated higher noise which would present an increasing challenge for regulatory mechanisms to maintain expression reliability.

### Between-copy transcript noise

Finally, simulations of transcription from separate centres within nuclei indicate that Kr plays a role in decreasing locus-to-locus (‘nuclear dot’) variability (Fig. 6). Such variability was recently investigated experimentally [19], demonstrating a high degree of noise and independence between loci within nuclei (and that different gap transcripts, including *hb* and *Kr*, showed similar concentrations and noise levels). These authors noted that the observed degree of noise smoothing from fresh transcripts to cytoplasmic levels in NC13 could be achieved by diffusion, but that cell membrane formation in NC14 could limit this. Our simulations indicate that Kr activation-inhibition of *hb* in mid-NC14 could be a means for smoothing between-dot noise during the cellularization process, particularly in the boundary region critical to PS4 and T2 formation. We predict that Kr effects on within nucleus transcript noise could be observed by comparing *hb* expression at nuclear dot resolution between WT and *Kr-* embryos in mid-NC14.

### Future directions

Fine-grained modelling, taking into account the TFBSs in enhancers, has been approached by a number of groups in recent years at a thermodynamic level, with transcription rates dependent on the steady-state fractional occupancy of TFBSs in the enhancers (e.g. [79,80], see review in [81]). These models are becoming increasingly effective in inferring expression patterns from regulatory sequences (e.g. [82]). The stochastic approach we have used is also fine-grained, but it is kinetic, modelling the time-course of BS-binding and transcription events. These kinetic simulations generate intrinsic noise distributions for mRNA and protein concentrations. For future directions, it is anticipated that regulatory frameworks inferred from thermodynamic models could be transformed into kinetic formulations in order to characterize noise propagation during gene expression. In addition, the use of automated parameter searches, used increasingly in thermodynamic and coarse-grained (e.g. network) models, can provide broader contexts for regulatory mechanisms. For example, in preliminary work on a related project, we have used an evolutionary computations optimization approach (e.g. see [83]) to search numerous potential Hb-Kr interactions for producing the PS4 stripe, and corroborated (at a coarse-grained/non-BS level) the same dual regulation, co-factor, cross-inhibition dynamics of the current model (Tables 1–3).

The robust development of an organism requires multiple levels of error correction. In segmentation patterning, the precision of maternal signalling gradients (particularly Bcd) has been extensively studied in recent years. Here, we focus on downstream events, during which gap-gap interactions become increasingly important in mid NC14 (the MBT), reducing the early NC14, maternally-dependent variability by half [12]. The **Kr dual** model, developed from quantitative experiments, indicates, when solved deterministically, how Hb and Kr co-regulate to form the refined PS4 stripe, and, when solved stochastically, how these Hb-Kr interactions generate precise expression in the mid-embryo, contributing to developmental robustness. Since MBT pattern refinement also occurs for the other gap and pair-rule genes, with similarly well-characterized cis-regulatory regions, the applicability of the dynamics characterized here for Hb-Kr is amenable to experimental and computational testing for other segmentation genes (e.g. with *giant* or *even-skipped* stripe formation, perhaps starting from the approach in [82]).

## Supporting Information

S1 Fig Simple Kr inhibition of *hb* produces unrefined anterior ‘step’ pattern. (PDF)

S2 Fig PS4 formation with the dual-dual mechanism. (PDF)

S3 Fig Interface noise reduction with mutual Hb-Kr interaction. (PDF)
