# Supplementary material for: Mid-Embryo Patterning and Precision in Drosophila Segmentation: Krüppel Dual Regulation of hunchback
Source: PLoS One. 2015 Mar 20;10(3):e0118450. doi: 10.1371/journal.pone.0118450 (PMC4368514; doi:10.1371/journal.pone.0118450)
Supplement: S1 Fig — (PDF) [file pone.0118450.s001.pdf]

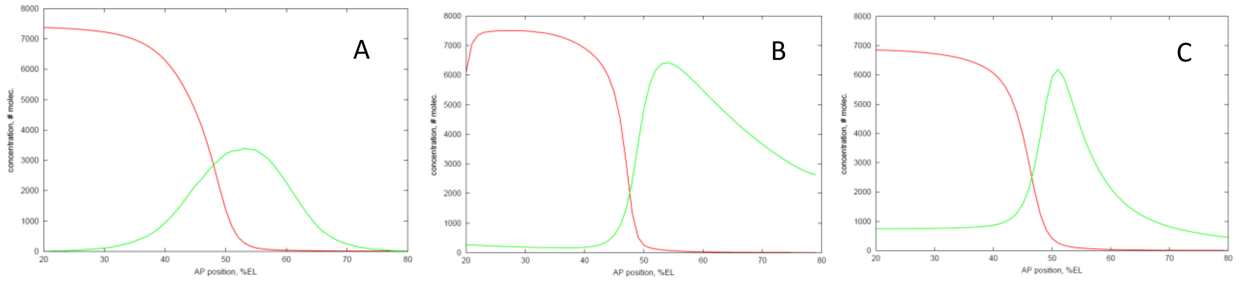

S1 Figure. Simple Kr inhibition of *hb* produces unrefined anterior ‘step’ pattern. (Kr only inhibits *hb*, it provides no activation.) Deterministic solutions of mechanisms: (**A**) static Kr; (**B**) Hb-Kr mutual inhibition (**mut inh**); and (**C**) **Hb dual**. Red – Hb protein; Green – Kr protein. Shown at *t*=30 minutes. Vertical, number of protein molecules per nucleus + surrounding cytoplasm; horizontal, AP position in %EL. (**A**) static Kr model: *hb* is activated by Bcd and Hb (as in [37]), Kr is a fixed, unchanging concentration gradient which inhibits *hb*. (**B**) mutual inhibition (**mut inh**): Kr is now dynamic – activated by Bcd and inhibited Hb; *hb* is activated by Bcd and Hb, and inhibited by Kr. (**C**) **Hb dual** model: Kr is dual regulated by Hb – activated at low [Hb] and inhibited at high [Hb] (Bcd activation of *Kr* is not modelled); Hb is as in (**A**), (**B**). In all cases, *hb* activation by Bcd and Hb and inhibition by Kr produces an unrefined anterior Hb step pattern.
