# Supplementary material for: Mid-Embryo Patterning and Precision in Drosophila Segmentation: Krüppel Dual Regulation of hunchback
Source: PLoS One. 2015 Mar 20;10(3):e0118450. doi: 10.1371/journal.pone.0118450 (PMC4368514; doi:10.1371/journal.pone.0118450)
Supplement: S2 Fig — (PDF) [file pone.0118450.s002.pdf]

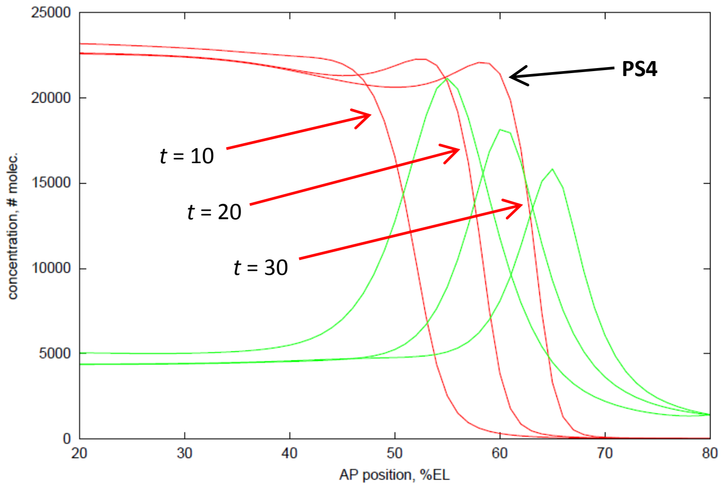

S2 Figure. PS4 formation with the dual-dual mechanism. Hb – red, Kr – green. Shown at *t*= 10, 20, 30 minutes into NC14. Hb dual regulates *Kr* (activating at low [Hb] and inhibiting at high [Hb]); and Kr dual regulates *hb* (activating at low [Kr] and inhibiting at high [Kr]). This forms the Hb PS4 peak, but the mutual activation-inhibition creates a posteriorly travelling boundary.
