# Supplementary material for: Mid-Embryo Patterning and Precision in Drosophila Segmentation: Krüppel Dual Regulation of hunchback
Source: PLoS One. 2015 Mar 20;10(3):e0118450. doi: 10.1371/journal.pone.0118450 (PMC4368514; doi:10.1371/journal.pone.0118450)
Supplement: S3 Fig — (PDF) [file pone.0118450.s003.pdf]

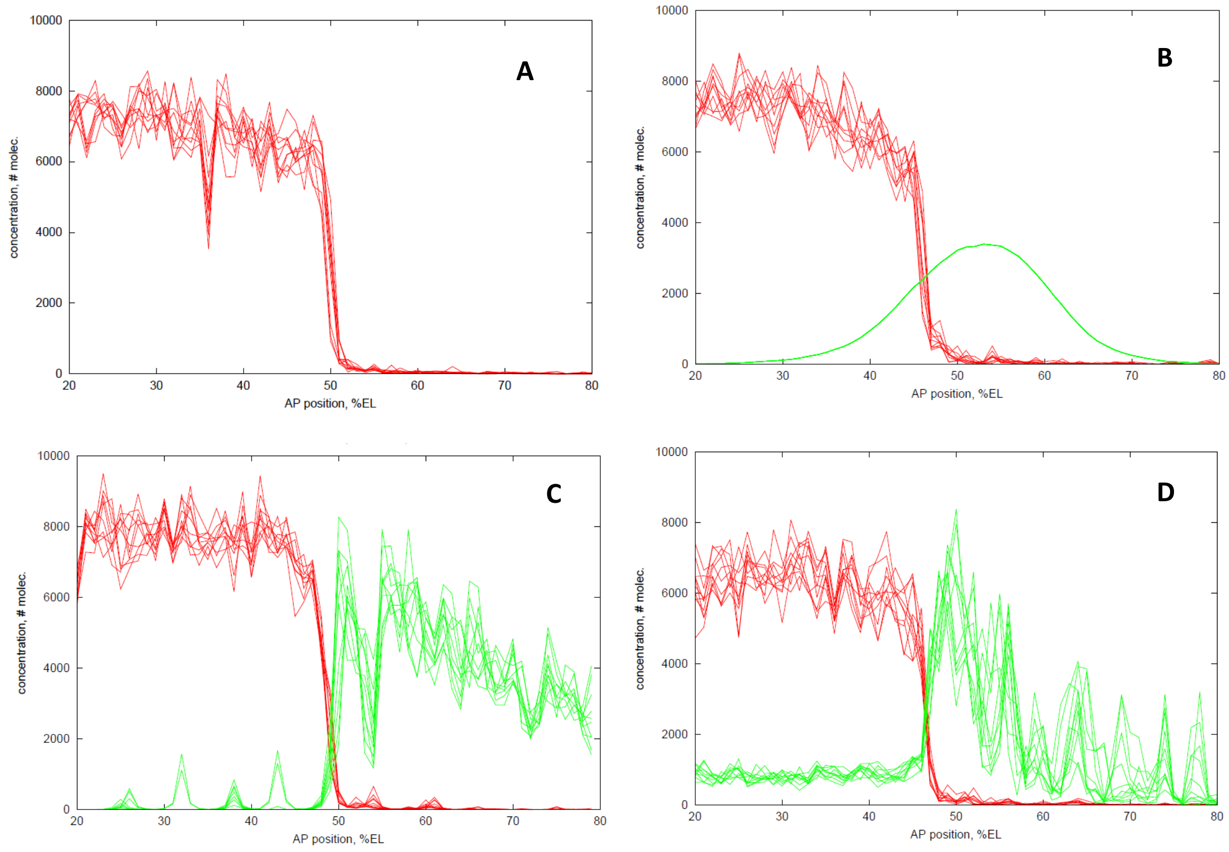

E

Hb range at half height	no Kr (A)	static Kr (B)	mutual inhibition (C)	Hb dual (D)
**mean**	1.10	1.05	0.55	0.50
**std. dev.**	0.37	0.63	0.25	0.24
n	11	8	10	10

S3 Figure. Interface noise reduction with mutual Hb-Kr interaction. Hb – red, Kr – green. All simulation results shown as overlays at 1 minute intervals, *t*=20 – 29. (**A**) no Kr, Hb expression depends on Bcd and Hb; (**B**) static Kr (Kr is not dynamically maintained, it is a fixed inhibition gradient for *hb*); (**C**) Hb-Kr mutual inhibition (**mut inh**, both are activated by Bcd and inhibit each other); (**D**) **Hb dual** mechanism (Hb activates and inhibits *Kr*, Kr simply inhibits *hb*). (**E**) Statistics for multiple simulations with each mechanism. Over *t*=20 – 29, the **mut inh** and **Hb dual** mechanisms have lower ranges in the position at which Hb crosses half-height than with no Kr or with static Kr (*t*-test of mean differences, $p<0.05$). (A), (B), (C), (D) show simulations with average range of position for each mechanism. This indicates that not only simple Kr inhibition of *hb* (B), but their mutual interaction, plays a role in making their interface more determinate.
